# Prophylactic Perioperative Sodium Bicarbonate to Prevent Acute Kidney Injury Following Open Heart Surgery: A Multicenter Double-Blinded Randomized Controlled Trial

**DOI:** 10.1371/journal.pmed.1001426

**Published:** 2013-04-16

**Authors:** Michael Haase, Anja Haase-Fielitz, Michael Plass, Hermann Kuppe, Roland Hetzer, Claire Hannon, Patrick T. Murray, Michael J. Bailey, Rinaldo Bellomo, Sean M. Bagshaw

**Affiliations:** 1 Department of Nephrology and Hypertension, Diabetes, and Endocrinology, Otto-von-Guericke-University Magdeburg, Germany; 2 Department of Anesthesiology, The German Heart Center, Berlin, Germany; 3 Department of Cardiothoracic Surgery, The German Heart Center, Berlin, Germany; 4 Nephrology and Clinical Pharmacology, University College Dublin School of Medicine and Medical Science, Dublin, Ireland; 5 The Australian and New Zealand Intensive Care Research Centre, Department of Epidemiology and Preventive Medicine, Monash University, Melbourne, Australia; 6 Department of Intensive Care, The Austin Hospital, Melbourne, Australia; 7 Division of Critical Care Medicine, Faculty of Medicine and Dentistry, University of Alberta, Alberta, Canada; University College London, United Kingdom

## Abstract

**Background:**

Preliminary evidence suggests a nephroprotective effect of urinary alkalinization in patients at risk of acute kidney injury. In this study, we tested whether prophylactic bicarbonate-based infusion reduces the incidence of acute kidney injury and tubular damage in patients undergoing open heart surgery.

**Methods and Findings:**

In a multicenter, double-blinded (patients, clinical and research personnel), randomized controlled trial we enrolled 350 adult patients undergoing open heart surgery with the use of cardiopulmonary bypass. At induction of anesthesia, patients received either 24 hours of intravenous infusion of sodium bicarbonate (5.1 mmol/kg) or sodium chloride (5.1 mmol/kg). The primary endpoint was the proportion of patients developing acute kidney injury. Secondary endpoints included the magnitude of acute tubular damage as measured by urinary neutrophil gelatinase-associated lipocalin (NGAL), initiation of acute renal replacement therapy, and mortality. The study was stopped early under recommendation of the Data Safety and Monitoring Committee because interim analysis suggested likely lack of efficacy and possible harm. Groups were non-significantly different at baseline except that a greater proportion of patients in the sodium bicarbonate group (66/174 [38%]) presented with preoperative chronic kidney disease compared to control (44/176 [25%]; *p* = 0.009). Sodium bicarbonate increased urinary pH (from 6.0 to 7.5, *p*<0.001). More patients receiving bicarbonate (83/174 [47.7%]) developed acute kidney injury compared with control patients (64/176 [36.4%], odds ratio [OR] 1.60 [95% CI 1.04–2.45]; unadjusted *p* = 0.032). After multivariable adjustment, a non-significant unfavorable group difference affecting patients receiving sodium bicarbonate was found for the primary endpoint (OR 1.45 [0.90–2.33], *p* = 0.120]). A greater postoperative increase in urinary NGAL in patients receiving bicarbonate infusion was observed compared to control patients (*p* = 0.011). The incidence of postoperative renal replacement therapy was similar but hospital mortality was increased in patients receiving sodium bicarbonate compared with control (11/174 [6.3%] versus 3/176 [1.7%], OR 3.89 [1.07–14.2], *p* = 0.031).

**Conclusions:**

Urinary alkalinization using sodium bicarbonate infusion was not found to reduce the incidence of acute kidney injury or attenuate tubular damage following open heart surgery; however, it was associated with a possible increase in mortality. On the basis of these findings we do not recommend the prophylactic use of sodium bicarbonate infusion to reduce the risk of acute kidney injury. Discontinuation of growing implementation of this therapy in this setting seems to be justified.

**Trial registration:**

ClinicalTrials.gov NCT00672334

*Please see later in the article for the Editors' Summary*

## Abstract 2

In a double-blinded randomized controlled trial, Anja Haase-Fielitz and colleagues find that an infusion of sodium bicarbonate during open heart surgery did not reduce the risk for acute kidney injury, compared with saline control.

## Editors' Summary

**Background:**

Open heart surgery is a type of cardiac surgery that is used to treat patients with severe heart disease, where the patient's chest is cut open and surgery is performed on the internal structures of the heart. During open heart surgery, surgeons may use a technique called cardiopulmonary bypass to temporarily take over the function of the heart and lungs. This type of surgery may be used to prevent heart attack or heart failure in patients with conditions such as angina, atherosclerosis, congenital heart disease, or valvular heart disease. There are a number of complications associated with open heart surgery and one of these is the rapid loss of kidney function, known as acute kidney injury (AKI), and formerly known as acute renal failure. Symptoms of AKI can be variable, with diagnosis of AKI based on laboratory findings (such as elevated blood urea nitrogen and creatinine), or clinical signs such as inability of the kidneys to produce sufficient amounts of urine. Globally, more than 10 million people are affected by AKI each year. AKI occurs in about one quarter of patients undergoing cardiac surgery and is associated with longer stays in the hospital and an increased risk of death. Treatment of AKI includes administration of intravenous fluids, diuretics, and, in severe cases, patients may require kidney dialysis.

**Why Was This Study Done?:**

The mechanism for why AKI occurs during cardiac surgery is complex and thought to involve multiple factors relating to blood circulation, the immune system, and toxins released by the kidneys. In addition to treating AKI after it occurs, it is important to identify patients who are at risk for developing AKI prior to cardiac surgery and then apply techniques to prevent AKI during cardiac surgery. A number of interventions have been tested for preventing AKI during cardiac surgery, but there is currently no strong evidence for a standard way to prevent AKI. One intervention that has potential for preventing AKI is the administration of sodium bicarbonate during cardiac surgery. Sodium bicarbonate causes alkalinization of the urine, and it is thought that this could reduce the effect of toxins in the kidneys. A previous pilot study showed promising effects for sodium bicarbonate to reduce the likelihood of AKI. In a follow-up to this pilot study, here the researchers have performed an international randomized controlled trial to test whether administration of sodium bicarbonate compared to sodium chloride (saline) during cardiac surgery can prevent AKI.

**What Did the Researchers Do and Find?:**

350 patients undergoing open heart surgery with at least one risk factor for developing AKI were recruited across four sites in different countries (Germany, Canada, Ireland, and Australia). These patients were randomly assigned to receive either sodium bicarbonate (treatment) or saline control solution, given as a continuous infusion into the blood stream for 24 hours during surgery. Neither the researchers nor the patients were aware of which patients were assigned to the treatment group. The researchers measured the occurrence of AKI within the first 5 days after surgery and they found that a greater proportion of those patients receiving sodium bicarbonate developed AKI, as compared to those patients receiving saline control. On the basis of these findings the study was terminated before planned recruitment was completed. A key issue with this study is that a greater proportion of the patients in the sodium bicarbonate group had chronic kidney disease prior to open heart surgery. After adjusting for this difference in the statistical analysis, the researchers observed that the difference between the groups was not significant—that is, it could have happened by chance. The authors also observed that a significantly greater proportion of patients receiving sodium bicarbonate died in the hospital after surgery compared to patients receiving saline control.

**What Do These Findings Mean?:**

These findings suggest that giving an infusion of sodium bicarbonate to induce alkalinization of the urine during open heart surgery is not a useful treatment for preventing AKI. Furthermore, this treatment may even increase the likelihood of death. The researchers do not recommend the use of sodium bicarbonate infusion to reduce the risk of AKI after open heart surgery and stress the need for discontinuation of this therapy. Key limitations of this research study are the early termination of the study and the greater proportion of patients with chronic kidney disease prior to surgery.

**Additional Information:**

Please access these Web sites via the online version of this summary at http://dx.doi.org/10.1371/journal.pmed.1001426.

- The Renal Association, a professional association for kidney doctors and researchers, provides information about acute kidney injury
- The International Society for Nephrology and the International Federation of Kidney Foundations provide information about preventing acute kidney injury around the world and jointly initiated World Kidney Day
- MedlinePlus has information on open heart surgery

## Introduction

Acute kidney injury is a global health problem with more than 10 million people affected annually [1]. It is independently related to long-term morbidity [2] and an estimated 4 million people die of acute kidney injury each year [1]. Evidence suggests that even minimal increases in serum creatinine are associated with poorer outcomes [3]. Acute kidney injury induces injury to distant organs such as the lungs, heart, and brain and therefore requires interdisciplinary care. Because of the known timing of the onset of renal injury, cardiac surgery is considered to be a clinically and scientifically highly relevant model of acute kidney injury. Also, cardiac surgery is one of the most frequent causes of acute kidney injury worldwide [4].

Although the pathophysiology of cardiac surgery-associated acute kidney injury is complex, poorly liganded iron appears to be linked to its development suggesting that acute kidney injury, may, in part, be a sideropathy [5]. Accordingly, a single center pilot randomized controlled trial enrolling a broad cohort of cardiac surgical patients recently reported a reduction in postoperative acute kidney injury from 52% to 32% (odds ratio [OR] 0.43) through perioperative urinary alkalinization with no significant side effects [6]. Administration of sodium bicarbonate was also associated with attenuation of postoperative acute tubular damage [6]. The mechanism behind these observed protective effects is thought to relate to the ability of bicarbonate to alkalinize the urine and to slow the Haber-Weiss reaction that generates reactive oxygen species via iron-dependent pathways [7]. Bicarbonate may also directly scavenge other reactive species, including hydroxyl radicals and peroxynitrite, from the blood [8]. These postulated mechanisms of action for sodium bicarbonate are supported by the finding from a large meta-analysis on contrast-nephropathy—another form of acute kidney injury where iron-related oxidative stress has been implicated in its pathophysiology—with only studies that achieved alkalinization of urine, demonstrating a positive outcome [9]. A recent review identified urinary alkalinization as the single most important drug-based intervention with convincing evidence to prevent acute kidney injury [10].

Given its biological rationale, a positive pilot trial, the broad discussion of the nephroprotective effects of bicarbonate infusion with positive risk benefit ratio, the lack of effective therapies, and the low costs, perioperative use of bicarbonate infusion has already impacted clinical practice of acute kidney injury prevention worldwide.

Accordingly, we conducted a multicenter double-blinded randomized controlled trial to confirm or refute whether prophylactic urinary alkalinization with perioperative sodium bicarbonate infusion reduces the incidence of acute kidney injury and the magnitude of acute tubular damage compared to volume- and solute-equivalent sodium chloride infusion in a larger patient population undergoing open heart surgery.

## Methods

### Design Overview

This study was an international, double-blinded, randomized controlled trial conducted at four university-affiliated hospitals (The German Heart Center Berlin, Germany; The Austin Hospital, Melbourne, Australia; The Mazankowski Alberta Health Institute, University of Alberta, Edmonton, Canada; and, The University College Dublin, Ireland; study protocol, Text S1). The Human Research Ethics Committee of each study center approved this study. Written informed consent was obtained from each patient before their surgery. The study adhered to the declaration of Helsinki and was reported following the recommendations of the CONSORT group [11] and its extension for reporting harms in randomized controlled trials (Text S2). The study was registered as “BIC-MC Study” with ClinicalTrials.gov. (http://clinicaltrials.gov/ct2/show/NCT00672334).

### Setting and Participants

Patients were eligible for inclusion if they were scheduled for elective or urgent open heart cardiac surgery. Inclusion and exclusion criteria are in line with literature for typical patient cohorts in this setting [6],[12],[13] and are shown in Box 1. Center-specific technical points during the conduction of cardiopulmonary bypass are shown in Table S1.

**Box 1. Inclusion and Exclusion Criteria**

**Inclusion criteria.** Cardiac surgical patients in whom the use of cardiopulmonary bypass was planned and having *one or more* of the following risk factors for postoperative acute kidney injury:

- Age above 70 y
- Pre-existing renal impairment (preoperative plasma creatinine concentration >120 µmol/l
- New York Heart Association class III/IV or impaired left ventricular function (left ventricular ejection fraction <35%)
- Valvular surgery or concomitant valvular and coronary artery bypass graft surgery
- Redo cardiac surgery
- Insulin-dependent Type 2 diabetes mellitus

**Exclusion criteria**

- End stage renal disease (serum creatinine concentration >300 µmol/l)
- Emergency cardiac surgery
- Planned off-pump cardiac surgery
- Known blood-borne infectious disease
- Chronic inflammatory disease on immunosuppression
- Chronic moderate to high dose corticosteroid therapy (>10 mg/d prednisone or equivalent)
- Enrolled in conflicting research study
- Age <18 y

### Randomization and Study-Related Interventions

Allocation concealment to patients, anesthesiologists, cardiac surgeons, intensive care specialists, bedside nurses, outcome assessors, and investigators was ensured by central randomization through a clinical trial pharmacist who was not a co-investigator (Department of Pharmacy) at each study center. At each study center, the hospital pharmacy clinical trials coordinator used a Microsoft Excel-based (Microsoft Corp.) random number generator to create the randomization list using a permuted block strategy with blocks of six. The randomization list was kept locked on a password-secured computer that was placed in the Hospital Pharmacy Trials Area. Infusion bags were each delivered in separate shrink-wrapped black plastic bags that were identical in appearance. At each study center, the responsible hospital trial pharmacist was provided with emptied study medication infusion bags to assess intactness of the covering bag (shrink-wrapped black plastic bag). In two cases, the covering bag was damaged. The inner bag (beneath the black bag) was a neutral infusion bag which was not labeled with direct information about the study treatment. Treatment allocation was only revealed after the study had been completed, the database locked, and statistical analysis completed. During the initiation period of the study we assessed whether blinding of study treatment was still in place by frequent discussion with clinical and research personnel and participants. These discussions happened the day after surgery. The general response was that there was sufficient uncertainty regarding doubtless treatment allocation precluding the need for elaborate activities on blinding status.

We used the same dosing of bolus of sodium bicarbonate and sodium chloride recently published in the prevention of cardiac surgery-associated acute kidney injury [6]. Study infusion bolus consisted of 0.5 mmol/kg of body weight in 250 ml given over 1 h commencing with induction of anesthesia. During the following 23 h continuous intravenous infusion of sodium bicarbonate or sodium chloride at a dose of 0.2 mmol/kg/h in 1,000 ml was administered. Bolus and continuous infusion achieved a total volume of 1.25 l and a total dose of 5.1 mmol/kg over 24 h, a 25% greater dosing regimen than that used in the pilot study [6]. The choice of control fluid (with the only difference being chloride anion instead of bicarbonate anion) was dictated by the need to deliver the same amount of sodium and the same amount of water in an approach that was similar to that applied in the pilot study.

Apart from the addition of the infusion of study drug, clinical practice was not changed or modified for the purpose of the study. For non-study-related interventions, please see Box 2.

**Box 2. Non-Study Related Interventions (Standard Perioperative Protocol at All Participating Centers)**

*ACE inhibitors/angiotensin receptor 1 blockers:*

- withdrawn on hospital admission (generally 1 d before surgery)

*Surgical approach and cardiopulmonary bypass:*

- conducted according to the standard technique of each institution

*Anesthetic techniques, including perioperative medications and fluid management:*

- at the discretion of the attending anesthetist (documented)

*Analgesia:*

- acetaminophen and morphine or tramadol with avoidance of non-steroidal anti-inflammatory drugs

*Vancomycin and gentamicin:*

- not used as perioperative anti-infective agents

*Post-operative care, including hemodynamic, fluid, and analgesic management:*

- at the discretion of the intensivists and nursing staff at each institution

### Outcomes and Follow-up

The primary endpoint was the number of patients who developed acute kidney injury. This was defined a priori as an increase in serum creatinine concentration greater than 25% or 0.5 mg/dl (44 µmol/l) from baseline to peak value at any time within the first 5 d after cardiopulmonary bypass. This endpoint has been used for previous studies of both cardiac surgery-associated acute kidney injury [13] and contrast-induced nephropathy [14] and was the same as used in the pilot study [6]. Secondary endpoints [15] and adverse events are shown in Box 3.

**Box 3. Secondary Outcomes and Adverse Events**

*Secondary renal outcomes:*

- changes of urinary NGAL concentration within the first 24 h after commencement of cardiopulmonary bypass were used to assess postoperative acute tubular damage as was previously done [6]
- Number of patients who developed acute kidney injury according to the RIFLE criteria [15] (using changes in creatinine and urinary output), number of patients with an increase in serum creatinine concentration >50% and >100%, and changes in serum creatinine and serum urea concentrations from baseline to peak value within the first 5–7 postoperative days
- Number of patients who received acute renal replacement therapy during their hospital stay

*Other secondary outcomes:*

- Changes in acid-base status
- Duration of mechanical ventilation and length of intensive care unit and hospital stay
- In-hospital mortality
- 90-d mortality

*Specific adverse events:*

- Postoperative incidence of hypernatremia ([Na^+^]>150 mmol/l), hypokalemia ([K^+^]<3.5 mmol/l), alkalemia (pH>7.50), acidemia (pH<7.30), atrial fibrillation, acute kidney injury, initiation of acute renal replacement therapy and mortality (in-hospital/at 90 d postoperatively) were recorded using the hospital electronic medical filing system or systematic telephone call of patients or their physicians at day 90

All data were prospectively collected and entered into a computerized database at each institution by blinded research nurses. At the completion of the study center-specific databases were combined prior to analysis. The combined database was checked for consistency from each center.

Serum creatinine and urea were measured before operation and at least daily during the first 7 d after open heart surgery. For measurement of neutrophil gelatinase-associated lipocalin (NGAL) on the ARCHITECT platform (Abbott Diagnostics) or ELISA (Antibodyshop), urine was sampled prior to induction of anesthesia, at 6 h and at 24 h after commencement of cardiopulmonary bypass. The laboratory investigators were blinded to the sample sources and clinical outcomes until the end of the study.

### Statistical Analysis

Using data from a recently published randomized controlled trial on the use of bicarbonate for acute kidney injury prevention in cardiac surgical patients [6], we estimated that 500 patients would be needed in this multicenter trial including a 10% loss to follow-up in the primary endpoint to have a >90% power to detect a 15% change in the proportion of patients receiving sodium bicarbonate who develop acute kidney injury (35%) as defined compared to control patients (50%) at a two-sided test with alpha of 0.05. The pilot study found an absolute acute kidney injury group difference of 20% (control 52%, bicarbonate 32%) [6]. For higher variation in outcome measures—which is inherent to multicenter studies—we conservatively anticipated this minimal clinically important difference in the development of acute kidney injury to be 15%. Interim analysis was intended to be performed at about 70% of planned patients by the Data Safety and Monitoring Committee. The Data Safety and Monitoring Committee was provided with enrolment data, subject disposition, baseline characteristics, protocol violations, adverse events, and outcomes and included acknowledged experts in the field. Stopping criteria were defined as follows: (i) larger than expected benefit, (ii) improbability for achieving a beneficial group difference for the primary endpoint, or (iii) recognizable harm of study treatment (significantly increased primary endpoint, renal replacement therapy or in-hospital mortality at *p*<0.05).

All following analyses were set a priori. All data were analyzed according to the intention-to-treat principle. In addition, the primary endpoint was analyzed per protocol. Per-protocol analysis excluded patients who were not operated on cardiopulmonary bypass or who were not drug-adherent defined as not receiving study infusion bolus before commencement of cardiopulmonary bypass or receiving <80% of the total weight-adjusted dose of study medication within the study period. Whilst there were insufficient events to facilitate multivariable analysis for mortality, to ensure all other observed results were not due to center or baseline imbalances, intention-to-treat analysis was repeated in multivariable models including all baseline variables that were considered to be clinically relevant or different between groups with univariate *p*<0.10. These variables were (i) clinically relevant (with some of them also being significantly different between groups in univariate analysis): age, cardiopulmonary bypass time, chronic obstructive pulmonary disease, chronic kidney disease (eGFR <60 ml/min) [16], peripheral vascular disease, and valve surgery and (ii) different between groups with univariate *p*<0.10: center, previous myocardial infarction, and red blood transfusion.

We also performed post hoc subgroup analyses including exclusion of the German study center, patients with eGFR >60 ml/min, and those receiving non-study-related bicarbonate infusion (see Table S2). Serum creatinine-based acute kidney injury diagnosis is influenced by several factors including its volume of distribution. We calculated postoperative cumulative fluid balance and computed a fluid-adjusted serum creatinine concentration [17] reflecting the effect of volume of distribution during the development phase of acute kidney injury. We used this fluid-adjusted serum creatinine concentration for recalculation of the number of patients developing the primary study endpoint.

Continuous data were examined for normal distribution using histograms. Between-group comparisons for continuous data were performed with the use of the Student's *t* test (specifically denoted where used) or the Mann-Whitney *U* test and for categorical data with the use of Fisher exact test or chi-square test where appropriate. All tests were two-tailed and we considered a *p*-value<0.05 to indicate statistical significance.

We report values as mean with standard deviation (SD), or median with 25th to 75th percentiles or as ORs with 95% CI estimate as appropriate. Analysis was performed using SPSS, version 18.0 (SPSS Inc.) and SAS version 9.2 (SAS Institute, Inc.).

## Results

### Patient Flow

**Figure 1:**
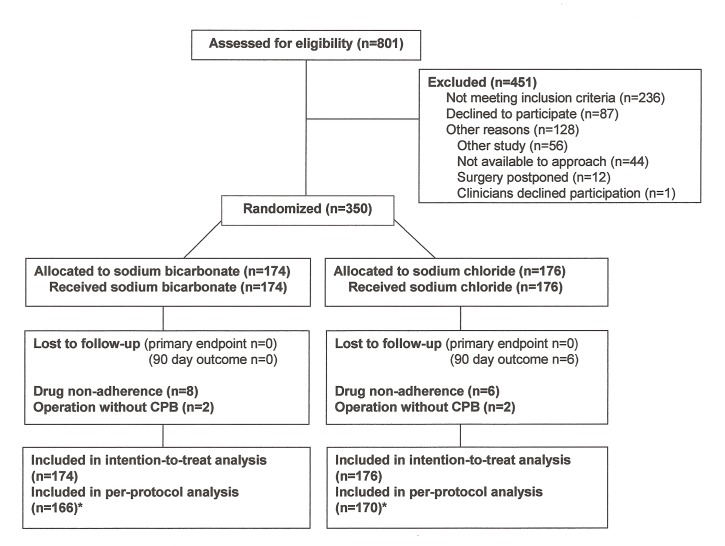
Patient flow through the study reported in a CONSORT [**11**] diagram. *Per protocol analysis excluded patients who were not operated on cardiopulmonary bypass or who were not drug-adherent. In both study groups there were two patients meeting both criteria. Six patients were lost to follow-up (all in the sodium chloride group). Major reasons for a patient to be lost to follow-up at 90 d were (i) patient came for open heart surgery from a foreign country and did not respond to contact attempts (*n* = 3) or (ii) patient was socially disadvantaged and did not provide a contact address or telephone number and did not visit their general practitioner or cardiac outpatient department for postoperative follow up (*n* = 3). CPB, cardiopulmonary bypass.

This study was a randomized controlled trial conducted at four university-affiliated hospitals (The German Heart Center Berlin, Germany [*n* = 200]; The Mazankowski Alberta Health Institute, University of Alberta, Edmonton, Canada [*n* = 98]; The Austin Hospital, Melbourne, Australia [*n* = 47] and, The University College Dublin, Ireland [*n* = 5]). Between May 2008 and June 2011, 350 patients were randomized to receive either intravenous sodium bicarbonate (*n* = 174) or sodium chloride (*n* = 176) infusion. Details of patients screened and participating in the study are shown in the CONSORT diagram (Figure 1). All patients received the assigned study treatment. The study was stopped early under recommendation of the Data Safety and Monitoring Committee because interim analysis suggested likely lack of efficacy and possible harm.

### Patient Characterization

Preoperative patient characteristics including number and type of inclusion criteria, exposure to nephrotoxins, and chronic medications were comparable between the treatment groups except that a greater proportion of patients in the bicarbonate group had chronic kidney disease (Tables 1 and 2). Patient groups were similar for preoperative median urinary NGAL concentration (bicarbonate: 3.7 [0.5–9.7] versus control: 4.3 [0.3–11.0]; *p* = 0.297). There was no significant difference between treatment groups in type and duration of operation, intraoperative hemodynamic variables, and interventions (Tables 3 and 4). Postoperative interventions including type and volume of administered fluids and use of vasopressors or inotropes and urinary output were similar in both groups (Table 5).

**Table 1:** Preoperative characteristics.

Characteristics	Sodium Bicarbonate (*n* = 174)	Sodium Chloride (*n* = 176)	p-Value
**Demographics**			
Age, y	66.4±12.1	64.6±13.5	0.190*
Male, *n*	125 (72%)	124 (71%)	0.745
Body mass index	27.5±4.4	27.5±5.7	0.999*
**Inclusion criteria**			
Age >70 y, *n*	80 (46%)	76 (43%)	0.599
Valve replacement, *n*	136 (78%)	141 (80%)	0.653
NYHA III or IV, *n*	33 (19%)	32 (18%)	0.851
Serum creatinine >120 µmol/l (>1.4 mg/dl), *n*	32 (18%)	23 (13%)	0.171
Insulin-dependent diabetes mellitus, *n*	10 (6%)	6 (3%)	0.295
Reoperation, *n*	37 (21%)	35 (20%)	0.957
**Number of inclusion criteria, ** ***n***	2.0 (1.0–3.0)	2.0 (1.0–2.0)	0.434
**Comorbidities**			
Arterial hypertension, *n*	122 (70%)	116 (66%)	0.399
Pulmonary hypertension, *n*	37 (21%)	36 (20%)	0.852
Peripheral vascular disease, *n*	39 (22%)	27 (15%)	0.096
Non-insulin-dependent diabetes mellitus, *n*	25 (14%)	30 (17%)	0.491
Hypercholesterolemia, *n*	108 (62%)	104 (59%)	0.554
Ejection fraction <35%, *n*	27 (16%)	22 (13%)	0.454
Ejection fraction, %	51.1±14.5	51.4±14.6	0.847*
Atrial fibrillation, *n*	44 (25%)	43 (24%)	0.853
Carotid disease, *n*	15 (9%)	11 (6%)	0.398
Myocardial infarction^a^, *n*	16 (9%)	8 (5%)	0.085
Stroke^a^, *n*	3 (2%)	4 (2%)	0.990
Chronic obstructive pulmonary disease, *n*	31 (18%)	19 (11%)	0.061
Current smoker, *n*	28 (16%)	23 (13%)	0.423
**Systolic/diastolic blood pressure, mmHg**	124.7±19.5/69.9±11.3	130.2±20.0/71.0±13.0	0.010* ^/^0.398*
**Medications**			
Beta-blocker, *n*	111 (64%)	103 (59%)	0.312
Calcium channel blocker, *n*	35 (20%)	46 (26%)	0.182
ACE-inhibitor or angiotensin blocker, *n*	80 (46%)	75 (43%)	0.526
Diuretics, *n*	85 (49%)	75 (43%)	0.242
Statins, *n*	89 (51%)	81 (46%)	0.337
Non-steroidal antiinflammatory drugs, *n*	3 (2%)	3 (2%)	0.989
Exposure to nephrotoxins^b^, *n*	25 (14%)	26 (15%)	0.897

For continuous variables, values denote mean ± standard deviation.

a Last 6 mo preoperatively.

b Including contrast media within 72 h preoperatively.

* *p*-Values according to Students *t* test or median (25th–75th percentiles) (Mann Whitney *U* test).

NYHA, New York Heart Association; ACE, angiotensin-converting-enzyme.

**Table 2:** Preoperative renal function.

Renal Function	Sodium Bicarbonate (n = 174)	Sodium Chloride (n = 176)	*p*-Value
Serum creatinine, µmol/l	89.2 (75.8–114.9)	84.0 (76.0–101.9)	0.090
eGFR, ml/min/1.73 m^2^	69.0 (52.3–88.6)	76.7 (59.1–88.2)	0.058
Chronic kidney disease^a^	66 (38%)	44 (25%)	0.009
Serum urea, mmol/l	5.7 (4.1–7.0)	5.5 (3.7–7.0)	0.407

For continuous variables, values denote median (25th–75th percentiles) (Mann Whitney *U* test]).

a eGFR <60 ml/min/1.73 m^2^ using the CKD-EPI formula [16].

eGFR, estimated glomerular filtration rate.

**Table 3:** Intraoperative characteristics.

Intraoperative Characteristics	Sodium Bicarbonate (*n* = 174)	Sodium Chloride (*n* = 176)	*p*-Value
Duration of cardiopulmonary bypass, min	131 (94–177)	121 (96–154)	0.348
Duration of aortic cross-clamp, min	80 (59–120)	79 (59–105)	0.565
*Type of operation*			
Valve repair/replacement, *n*	79 (45%)	89 (51%)	0.333
CABG, *n*	31 (18%)	32 (18%)	0.929
Valve and CABG (concomitant), *n*	37 (21%)	35 (20%)	0.750
Thoracic aorta, *n*	23 (13%)	20 (11%)	0.597
Ventricular assist devices, *n*	2 (1%)	0 (0%)	0.246
Urgent operation^a^ *n*	50 (29%)	49 (28%)	0.853
Urine output, ml	1,340 (900–1940)	1,350 (940–1970)	0.701
Drainage, ml	100 (50–200)	150 (50–230)	0.170
Lowest mean arterial pressure, mmHg	43.5±16.0	45.0±16.0	0.381*
Cardiac index, l/min	2.3±0.7	2.4±0.7	0.851*
Intraoperative hemofiltration, *n*	31 (18%)	25 (14%)	0.357

For continuous variables, values denote mean ± standard deviation.

a Within 72 h after first cardiac symptoms.

* *p*-values according to Students t-test) or median (25th–75th percentiles) (Mann Whitney U test).

CABG, coronary artery bypass grafting.

**Table 4:** Intraoperative fluids and medications.

Intraoperative Fluids and Medications	Sodium Bicarbonate (*n* = 174)	Sodium Chloride (*n* = 176)	*p*-Value
**Crystalloids, ml**	2,310 (1,330–3,260)	2,350 (1,470–3,310)	0.499
**Colloids, ml**	200 (0–500)	500 (0–500)	0.485
**Packed red blood cells**			
Proportion of patients, *n*	66 (38%)	59 (34%)	0.389
Volume in patients with transfusion, ml	500 (500–750)	500 (350–650)	0.019
**Fresh frozen plasma**			
Proportion of patients, *n*	54 (31%)	55 (31%)	0.965
Volume in patients with transfusion, ml	880 (660–1320)	880 (660–1320)	0.956
**Platelets**			
Proportion of patients, *n*	14 (8%)	22 (12.5%)	0.170
Volume in patients with transfusion, ml	275 (150–360)	300 (170–430)	0.432
**Cryoprecipitate**			
Proportion of patients, *n*	2 (1%)	3 (2%)	0.999
Volume in patients with transfusion, ml	210 (150–170)	180 (80–350)	0.800
**Potassium substitution**			
Proportion of patients, *n*	140 (80.5%)	127 (72%)	0.068
Dose in patients with potassium, mmol/l	29 (18–50)	20 (11–40)	0.071
**Furosemide**			
Proportion of patients, *n*	43 (25%)	42 (24%)	0.853
Dose in patients with furosemide, mg	20 (20–40)	20 (10–20)	0.581

For continuous variables, values denote median (25th–75th percentiles) (Mann Whitney *U* test).

**Table 5:** Postoperative interventions.

Postoperative Interventions	Sodium Bicarbonate (*n* = 174)	Sodium Chloride (*n* = 176)	*p*-Value
**Norepinephrine**			
Day of surgery, *n*	129 (74%)	119 (68%)	0.179
Day 1 after surgery, *n*	40 (23%)	28 (16%)	0.094
**Epinephrine** a			
Day of surgery, *n*	73 (42%)	69 (39%)	0.600
Day 1 after surgery, *n*	57 (33%)	46 (26%)	0.174
**Inotropes** b			
Day of surgery, *n*	89 (51%)	81 (46%)	0.337
Day 1 after surgery, *n*	67 (39%)	60 (34%)	0.390
**Intraaortic balloon pump**			
Day of surgery, *n*	10 (6%)	5 (3%)	0.198
Day 1 after surgery, *n*	12 (7%)	5 (3%)	0.087
**Back to operation room**			
Day of surgery, *n*	5 (3%)	4 (2%)	0.750
Day 1 after surgery, *n*	5 (3%)	5 (3%)	0.999
**6–24 h**			
Crystalloids, ml	4,250 (2,860–6,010)	4,290 (2,980–5,840)	0.630
Colloids, ml	0 (0–200)	0 (0–200)	0.906
Urine output, ml	2,700 (1,680–4,020)	2,870 (1,840–3,690)	0.863
Drainage, ml	370 (270–600)	325 (225–530)	0.096
PRBC (patients with transfusion), ml	500 (350–840)	500 (500–990)	0.808
**24–48 h**			
Crystalloids, ml	3,000 (1,150–4,200)	3,000 (1,600–4,000)	0.973
Colloids, ml	0 (0–0)	0 (0–0)	0.930
Urine output, ml	2,700 (1,300–4,000)	2,700 (1,200–4,000)	0.974
Drainage, ml	250 (125–445)	230 (100–450)	0.671
PRBC (patients with transfusion), ml	500 (270–550)	500 (500–630)	0.582

For continuous variables, values denote median (25th–75th percentiles) (Mann Whitney *U* test).

a Refers to use of epinephrine as inotropic agent.

b Refers to use of epinephrine, dobutamine, and milrinone.

PRBC, packed red blood cells.

Drug adherence was 95.4% (166/174) for patients receiving bicarbonate and 96.6% (170/176) for patients receiving chloride study infusion, *p* = 0.570. Median total dose of sodium bicarbonate was 416 (362–455) mmol and 408 (349–474) mmol for sodium chloride, *p* = 0.652, both administered within the 24-h study period.

### Urinary pH and Plasma Biochemical Changes

Prior to induction of anesthesia, patients did not significantly differ in urinary pH and plasma biochemical variables (Table 6). Sodium bicarbonate infusion induced urinary alkalinization during the 24-h study intervention (*p*<0.001) whereas urinary pH level decreased in control patients (Table 6). Also, we found group differences in plasma concentrations of pH, bicarbonate, base excess, and potassium (Table 6).

**Table 6:** Changes in urinary pH and plasma biochemical variables.

Urinary pH and Plasma Biochemical Variables	Sodium Bicarbonate (*n* = 174)	Sodium Chloride (*n* = 176)	*p*-Value
**Urinary pH**			
Preoperative	6.0 (5.0–6.5)	6.0 (5.0–6.5)	0.094
6 h after CPB-Start	6.5 (5.5–7.0)	6.0 (5.0–6.0)	<0.001
24 h after CPB-Start	7.5 (6.1–8.0)	5.5 (5.0–6.5)	<0.001
**Plasma**			
pH			
Preoperative	7.40 (7.37–7.43)	7.41 (7.38–7.44)	0.368
6 h after CPB-Start	7.42 (7.36–7.42)	7.37 (7.33–7.43)	<0.001
24 h after CPB-Start	7.45 (7.42–7.47)	7.39 (7.36–7.42)	<0.001
Hemoglobin, g/dl			
Preoperative	12.5 (11.3–13.4)	12.6 (11.3–14.0)	0.237
6 h after CPB-Start	10.7 (9.4–11.5)	10.8 (9.5–11.7)	0.369
24 h after CPB-Start	10.0 (9.2–11.0)	10.5 (9.4–11.5)	0.020
Bicarbonate, mmol/l			
Preoperative	24.2 (22.6–25.7)	24.0 (22.6–25.7)	0.955
6 h after CPB-Start	25.3 (22.9–27.0)	22.9 (20.7–24.0)	<0.001
24 h after CPB-Start	30.0 (28.0–36.0)	24.2 (22.6–26.0)	<0.001
Base excess			
Preoperative	−0.1 (−1.5 to 1.2)	0 (−1.5 to 1.4)	0.431
6 h after CPB-Start	1.4 (−1.3 to 3.1)	−1.9 (−3.2 to −0.2)	<0.001
24 h after CPB-Start	5.9 (4.2–8)	−0.4 (−1.9 to 1.2)	<0.001
Lactate, mmol/l			
Preoperative	0.7 (0.6–1.0)	0.8 (0.6–1.0)	0.288
6 h after CPB-Start	1.7 (1.2–2.9)	1.5 (1.1–2.7)	0.243
24 h after CPB-Start	1.7 (1.2–2.6)	1.6 (1.2–2.3)	0.843
Potassium, mmol/l			
Preoperative	3.8 (3.6–4.1)	3.8 (3.6–4.1)	0.300
6 h after CPB-Start	4.1 (3.8–4.4)	4.3 (4.0–4.6)	0.003
24 h after CPB-Start	4.4 (4.0–4.8)	4.5 (4.1–4.8)	0.315
Sodium, mmol/l			
Preoperative	139 (137–140)	138 (137–140)	0.448
6 h after CPB-Start	140 (138–143)	140 (138–142)	0.958
24 h after CPB-Start	142 (139–145)	142 (139–145)	0.808

For continuous variables, values denote median (25th–75th percentiles) (Mann Whitney *U* test).

CPB, cardiopulmonary bypass.

### Primary Endpoint

**Figure 2:**
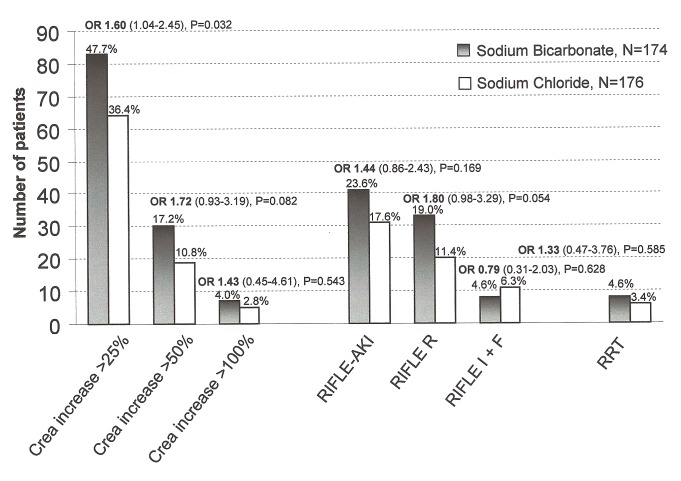
Renal endpoints for patients receiving sodium bicarbonate versus sodium chloride. Number of patients receiving sodium bicarbonate (black bars) developing acute kidney injury after open heart surgery compared to patients receiving sodium chloride (white bars). The OR (with 95% CI) shows the risk of developing a pre-defined renal endpoint of patients treated with sodium bicarbonate compared to those treated with sodium chloride. From left to right: primary endpoint: increase in plasma creatinine >25% or >0.5 mg/dl (>44 µmol/l), secondary endpoints: increase in plasma creatinine >50%, increase in plasma creatinine >100%, RIFLE-classification based acute kidney injury (risk, injury, failure loss, and end stage renal failure criteria, [15]) and acute renal replacement therapy (RRT) within index hospitalization.

In intention-to-treat analysis, a greater proportion of patients in the bicarbonate group developed the primary endpoint of acute kidney injury (83 [47.7%] versus 64 [36.4%]; OR 1.60 [95% CI 1.04–2.45]; unadjusted *p* = 0.032) compared with the control group (Figure 2).

After multivariable adjustment for group imbalances at baseline (for model see “Statistical Analysis”), a non-significant group difference was found for the primary endpoint (adjusted *p* = 0.120, OR 1.45 [0.90–2.33]).

Also, after adjustment of peak creatinine for the dilutional effect of corresponding fluid-balance, no significant group difference for the primary endpoint was found (sodium bicarbonate: 89/174 [51.1%] versus sodium chloride infusion: 76/176 [43.2%]; OR 1.38 [0.90–2.10], *p* = 0.135).

Per-protocol analysis of the primary endpoint including 166 patients receiving bicarbonate infusion and 170 patients receiving sodium chloride infusion (Figure 1), showed a higher incidence of AKI in the bicarbonate group compared with the control group (78/166 [47.0%] versus 60/170 [35.3%], OR 1.63 [1.05–2.52]; *p* = 0.029).

### Secondary Renal Endpoints

A greater median postoperative increase in urinary NGAL from preoperative to peak value within the first 24 h postoperatively in patients receiving bicarbonate infusion (24.3 ng/ml [6.0–149.9]) indicated more pronounced acute tubular damage after surgery compared to control patients (12.2 ng/ml [0.0–76.6]), *p* = 0.011.

**Figure 3:**
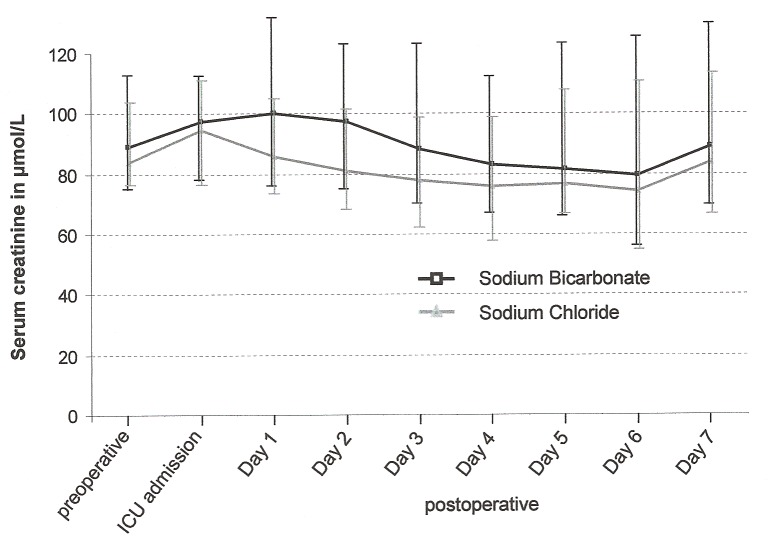
Serum creatinine in patients receiving sodium bicarbonate (black squares) or sodium chloride (grey triangles) infusion. Serum creatinine concentrations are shown over time (median [25th–75th percentiles]).

Non-significantly more patients in the bicarbonate group developed other renal endpoints including differential creatinine increase, acute kidney injury according to the Risk, Injury, Failure Loss, and End stage (RIFLE) classification [15] and the need for initiation of acute postoperative renal replacement therapy (Figure 2). Figure 3 shows the course of serum creatinine (without adjustment for dilutional effects) over time separated by treatment groups. Patients receiving bicarbonate developed a greater absolute creatinine increase (*p* = 0.015), higher peak serum creatinine (*p* = 0.001), and higher peak serum urea (*p* = 0.020) (Table 7).

**Table 7:** Outcomes.

Outcomes	Sodium Bicarbonate (*n* = 174)	Sodium Chloride (*n* = 176)	Odds Ratio (95% CI)	*p*-Value
**Renal outcomes**				
Δ Serum creatinine^a^, µmol/l	22 (8–44)	15 (5–29)	—	0.015
Peak serum creatinine^b^, µmol/l	106 (85–139)	95 (78–115)	—	0.001
Δ Serum urea^a^, mmol/l	2.4 (0.4–6.2)	1.9 (0.2–5.0)	—	0.117
Peak serum urea^b^, mmol/l	8.2 (6.2–12.3)	7.6 (5.4–10.0)	—	0.020
**Other outcomes**				
Length of ventilation, h	18 (13–26)	17 (13–26)	—	0.682
Length of stay in intensive care, h	33 (23–96)	28 (22–69)	—	0.255
Length of stay in hospital, days	17 (7–25)	18 (7–25)	—	0.996
New onset atrial fibrillation^c^	21 (12.1%)	20 (11.4%)	1.07 (0.56–2.05)	0.873
Readmission to hospital within 90 d, *n*	11 (6.3%)	16 (9.1%)	0.68 (0.30–1.50)	0.294
Died in hospital, *n*	11 (6.3%)	3 (1.7%)	3.89 (1.07–14.20)	0.031
Died after discharge within 90 d, *n*	2 (1.2%)	2 (1.1%)	1.01 (0.14–7.26)	0.999
Overall mortality within 90 d, *n*	13 (7.5%)	5 (2.8%)	2.76 (0.96–7.92)	0.056

For continuous variables, values denote median (25th–75th percentiles) (Mann Whitney *U* test).

a From preoperative value to postoperative peak within the first 5 postoperative days.

b Highest value within the first 5 postoperative days.

c Within 48 h postoperatively.

### Other Secondary Endpoints

We found no group differences in other clinical endpoints (Box 3) except for in-hospital mortality (Table 7). Eleven patients in the bicarbonate group and three in the control group died within the index hospital admission, *p* = 0.031 (Table 7), all outside the study treatment period. The majority of patients died after the first postoperative week (Table 8). Causes and time of death are also summarized in Table 8.

**Table 8:** Cause of in-hospital death.

Patient Number	Age, Sex	Type of Operation	Cause of Death	Postoperative Day	On ICU/After ICU	Treatment
2	83, F	Valve surgery	Cardiogenic shock	69	±	Bicarbonate
72	74, M	Thoracic surgery with aortic valve replacement	Myocardial re-infarction, ventricular fibrillation	21	±	Bicarbonate
73	53, F	Valve surgery	Cardiogenic shock, AKI (RRT)	16	±	Bicarbonate
107	65, F	Valve surgery	Ventricular fibrillation, cardiogenic shock, AKI	15	±	Bicarbonate
108	77, F	Valve surgery	Cardiogenic shock, AKI	6	±	Bicarbonate
115	69, M	Concomitant surgery (valve+4× CABG)	Cardiogenic shock, AKI	7	±	Control
122	77, F	Concomitant surgery (valve+single-bypass)	AV-block, CPR, bleeding, cardiogenic shock, AKI	2	±	Bicarbonate
165	84, F	Concomitant surgery (valve+single-bypass)	Myocardial re-infarction, ventricular fibrillation	48	±	Bicarbonate
173	75, F	Valve surgery	Hemolysis, ventricular fibrillation, cardiogenic shock AKI (RRT)	4	±	Control
177	64, F	Valve surgery	Cardiogenic shock	2	±	Bicarbonate
186	79, F	Thoracic surgery with aortic valve replacement	Severe coagulopathy, bleeding, RRT	35	±	Bicarbonate
191	77, M	Concomitant surgery (valve+2× CABG)	Cardiogenic shock, severe coagulopathy, bleeding, AKI (RRT)	16	±	Bicarbonate
203	71, F	4× CABG	Sepsis, AKI	23	±	Control
243	73, M	Ventricular assist device	Pneumonia, sepsis, AKI (RRT)	77	±	Bicarbonate

AKI, acute kidney injury (RIFLE criteria [15]); AV, atrio-ventricular; CABG, coronary artery bypass grafting; CPR, cardiopulmonary resuscitation; F, female; ICU, intensive care unit; M, male; RRT, renal replacement therapy.

Overall mortality within 90 d was 7.5% (*n* = 13) in the bicarbonate group and 2.8% (*n* = 5) in the control group; *p* = 0.056 (Table 7). In a sensitivity analysis of this endpoint, assuming that the six patients who were lost to follow-up (all in the control group) would have died within 90 d, the result remained non-significantly different (bicarbonate group: 7.5% [*n* = 13] versus control group: 6.3% [*n* = 11]); *p* = 0.651.

Center-specific analysis of the primary and all secondary endpoints is shown in Table S3.

### Subgroup Analyses for Major Endpoints

Subgroup analyses found non-significant group differences for renal outcomes, length of stay in hospital, and in-hospital mortality in (i) patients with preoperative chronic kidney disease and (ii) patients from the Australian, Canadian, and Irish study centers (Table S2). The latter analysis was performed, as patients from the German study center presented with more comorbidities, stayed longer in hospital, and died more frequently (13/200, total: 14/350 patients).

Analysis of patients excluding those with non-study bicarbonate infusion showed essentially unchanged major endpoints (Table S2).

### Safety

Four patients developed hypernatremia during the study period with peak sodium concentration of 151, 152, 154, 158 mmol/l, respectively. Thirteen patients in the control group and 37 patients in the bicarbonate group had a pH level greater than 7.50 with a peak value of 7.59 in both groups (*p*<0.001). Thirty patients in the control group and 14 patients in the bicarbonate group had a pH level below 7.30 with a minimum value of 7.17 in both groups (*p* = 0.011). Non-significantly more patients receiving sodium bicarbonate (14/174, 8.0%) had a plasma potassium <3.5 mmol/l compared to control (9/176, 5.1%) with the lowest potassium value of 3.0 mmol/l in both groups, *p* = 0.280. There was no significant difference in the incidence of new-onset atrial fibrillation (21/174, 12.1% versus 20/176, 11.4%), *p* = 0.838.

## Discussion

We conducted a multicenter, double-blinded, randomized controlled trial of bicarbonate infusion with the aim of testing its ability to decrease the incidence and severity of acute kidney injury. In this study, we tested a novel pathophysiological concept considering acute kidney injury being a potential sideropathy with oxidoinflammatory stress as a common unifying pathway causing cell injury aggravated by labile iron compounds [5]. We found that bicarbonate infusion achieved plasma and urinary alkalinization but did not reduce kidney function deterioration indicated by serum creatinine concentration and urinary output and did not attenuate acute tubular damage as measured by urinary NGAL concentration. Furthermore, several secondary outcomes suggest that bicarbonate infusion may have induced harm.

The Kidney Disease: Improving Global Outcomes (KDIGO) Group identified acute kidney injury to be a potentially preventable complication and recommended definitive randomized controlled trials for evaluating promising interventions to prevent and mitigate acute kidney injury [18]. A previous single center randomized pilot study demonstrated a lower incidence of acute kidney injury after cardiac surgery in patients who received sodium bicarbonate, administered in a similar dose and timing as used in this study [6]. In this pilot study [6], bicarbonate attenuated the post-operative increase in serum creatinine and urinary NGAL, with the latter being interpreted as a signal for reduced iron-related tubulotoxic oxidative stress [19]. This nephroprotective effect was not confirmed in this multicenter study. The similar patient demographics and concomitant treatment in the centers involved makes it possible that the observed benefit in the pilot study was due to a type I error [6]. The results of our multicenter trial are supported by a retrospective observational study showing no renal advantage of bicarbonate- over chloride-based infusion regimens in cardiac surgery patients [20].

In the present study, using the same scoring system [12] as used in both the pilot study [6] and other studies of this disorder [21], a cohort of patients at increased risk of developing acute kidney injury was identified with 42% developing the primary endpoint of a >25%/>0.5 mg/dl (>44 µmol/l) creatinine increase compared to 42% in the pilot trial [6]. The clinically relevant and statistically significant increase of urinary pH in patients receiving study bicarbonate infusion—consistent with the proposed mechanism of action—was similar to that observed in the pilot study.

This study demonstrates that there is no reduction of acute kidney injury or tubular protection after open heart surgery in patients who are administered sodium bicarbonate despite achieving adequate plasma and urinary alkalinization. This finding challenges the theory of attenuation of urinary mis-liganded iron compounds to be achieved by systemic alkalinization with bicarbonate. Experimental and clinical trials testing the effect of local urinary alkalinization without blood alkalinization—usually achieved by carbonic anhydrase inhibitors—are needed. We cannot exclude that perioperative systemic application of sodium bicarbonate and alkalinization of the blood had negative effects on unadjusted hospital mortality. There were insufficient events to facilitate multivariable analysis for in-hospital mortality, to ensure observed results were not due to center or baseline imbalances specifically given the group difference for preoperative chronic kidney disease. Despite the fact that the majority of patients died later in the course and no relationship of study bicarbonate infusion with mortality in the pilot study was observed [6], a causal association of systemically administered sodium bicarbonate and mortality appears to be possible.

Given our findings, we do not recommend the routine prophylactic use of sodium bicarbonate infusion for the purpose of urinary alkalinization and acute kidney injury prevention in patients undergoing open heart surgery, nor can we recommend conduct of larger scale clinical research in this setting. Using a preoperative risk stratification scoring system [12], we confirmed that it is feasible to accurately enroll a large subgroup of cardiac surgical patients at high risk of developing postoperative acute kidney injury with reproducible rates of acute kidney injury. We would recommend the use of similar inclusion criteria in future studies investigating acute kidney injury in this patient group. For the first time, we demonstrate the theragnostic value of an acute tubular damage marker, NGAL, in a multicenter acute kidney injury-prevention trial. Although the results of the present study were non-favorable for sodium bicarbonate infusion compared with the signal for potential nephroprotection reported in the pilot trial [6], urinary NGAL proved early to indicate acute tubular damage (within the first 6–24 h postoperatively) as previously reported [22]. Such a finding makes this biomarker interesting for use as a surveillance marker of therapeutic effects or as a study endpoint in further interventional trials directed at acute kidney injury. After an injurious event to the kidney, NGAL is released to large extent from the distal tubular epithelial cells within hours, while serum creatinine accumulates in the blood over days when glomerular filtration declines [23]. Recently, NGAL has been suggested to be an early indication of renal tubular stress and damage induced by ischemia-reperfusion, inflammation, or nephrotoxins [23]. The magnitude of NGAL increase correlates with that of renal function loss linking acute tubular damage occurring early in the course of acute kidney injury with subsequent renal function loss possibly mediated by tubulo-glomerular feedback mechanisms [24].

Given the positive effect of bicarbonate in the pilot trial and the negative effect in this randomized controlled multicenter trial, for sample size estimation in future preventive trials, we recommend using a more severe acute kidney injury endpoint definition such as doubling of serum creatinine or commencement of postoperative renal replacement therapy or a combination thereof. Such endpoints may decrease the chance of type I errors and may increase the likelihood of successful phase IIb trials. Finally, mechanistic studies linking alkalotic blood pH with organ damage are needed.

### Study Limitations

The timing and dose of bicarbonate bolus and the duration of bicarbonate maintenance infusion may have been insufficient. However, study medication was administered using exactly the same infusion protocol with a slightly increased dose of maintenance infusion compared to the previous successful pilot trial [6]. We cannot exclude that further increased urinary or blood pH at 24 h postoperatively may have had detrimental effects on patient outcomes.

Most patients were enrolled in one study center and a greater proportion of patients receiving sodium bicarbonate presented with preoperative chronic kidney disease compared to control; however, (i) multivariable, (ii) center-specific, and (iii) subgroup analyses revealed no nephroprotective effect of sodium bicarbonate. The study was prematurely terminated because of lack of efficacy and suggestion of harm; however, excellent study protocol adherence, no significant loss to follow-up, the use of kidney functional and acute tubular damage markers, and the use of preset sensitivity analyses strengthen the findings and the observation of possible harm make the likelihood of a positive finding implausible. Also, the internal and external validity of the results was achieved by double-blinding and a multicenter international design. Given the above observations, it appears unlikely that the negative outcome of this trial is due to type II error. We were not able to control for intentionally unblinding of study treatment by directly measuring pH value of the study treatment solution. Clinicians may have been able to guess group assignment on the basis of urinary and plasma pH levels being more alkaline in the treatment group after surgery (significant differences at 6 h and 24 h after surgery). However, assessment of such an attempt during study initiation for the day after surgery showed sufficient doubt, since there was some overlap in these values between the groups.

In conclusion, this study demonstrates that in patients at high risk of acute kidney injury following open heart surgery, bicarbonate infusion alkalinized both blood and urine but did not result in a decrease in the incidence of acute kidney injury or attenuation of acute tubular damage. Importantly, prophylactic bicarbonate might possibly have increased mortality. On this basis of our findings we do not recommend the prophylactic use of perioperative infusions of sodium bicarbonate to reduce the incidence or severity of acute kidney injury in this patient group. Accordingly, discontinuation of growing implementation of this intervention in this setting would appear justified.

## Supporting Information

Table S1 Center-specific management of cardiopulmonary bypass and perioperative hemodynamic management.
(DOC)

Table S2 Major endpoints in post hoc subgroup analysis.
(DOC)

Table S3 Outcomes separated by study center.
(DOC)

Text S1 Trial protocol.
(DOC)

Text S2 CONSORT checklist [11].
(DOC)

## Abbreviations

NGAL: neutrophil gelatinase-associated lipocalin
OR: odds ratio
