# Supplementary material for: Prophylactic Perioperative Sodium Bicarbonate to Prevent Acute Kidney Injury Following Open Heart Surgery: A Multicenter Double-Blinded Randomized Controlled Trial
Source: PLoS Med. 2013 Apr 16;10(4):e1001426. doi: 10.1371/journal.pmed.1001426 (PMC3627643; doi:10.1371/journal.pmed.1001426)
Supplement: Table S1 — Center-specific management of cardiopulmonary bypass and perioperative hemodynamic management. (DOC) [file pmed.1001426.s001.doc]

| **Table S1.** Center-specific management of cardiopulmonary bypass and perioperative hemodynamic management | | | | |
| --- | --- | --- | --- | --- |
| **CPB management** | **Center 1 (Berlin)** | **Center 2 (Edmonton)** | **Center 3 (Melbourne)** | **Center 4 (Dublin)** |
| Cooling protocol | - Cooling to mild hypothermia (34-35°C, heat exchanger [Stöckert, Germany])  - deep hypothermic circulatory arrest in patients with complex operation (aortic arch) => active cooling to 18-22°C  - (slow) active rewarming afterwards to 37°C | - Cooling is generally passive down to 33°C  - Exception: patient is to receive deep hypothermic circulatory arrest => active cooling to 18-22°C  - Temperature measured with a urine catheter probe  - active rewarming afterwards to 37°C | - Cooling to mild hypothermia (34-35°C, heat exchanger [Stöckert, Germany])  - active rewarming afterwards to 37°C  - Temperature measured with a urine catheter probe | - Cooling to mild  hypothermia (32-34°C)  -active rewarming  afterwards to 37°C  - Temperature measured  with a urine catheter probe |
| Antibiotics | - Cefazolin 1g iv or  Cefuroxim 1,5g iv  - Penicilline allergy: cephalosporine 2.generation (see above) or Clindamycin 600 mg iv | - Cefazolin 1g iv  - second-line alternatives for penicillin-allergic patients include Cefuroxime  - for patients with severe allergy against penicilline/ cephalosporins: Clindamycin 600 mg iv | - Cephalothin 1g iv  - Clindamycin 600 mg iv for patients with severe allergy against penicilline/ cephalosporins | - Cefazolin 1g iv  - Patients with severe  allergy against penicilline/ cephalosporins: third-generation cephalosporin (Ceftriaxone),  Clindamycin or Carbapenem |
| Duration of cardiopulmonary bypass, min | 121 (93-145) | 130 (100-177) | 131 (100-215) | 80 (77-88) |
| Duration of aortic cross-clamp, min | 73 (55-95) | 96 (66-133) | 98 (73-164) | 61 (57-64) |
| Packed red blood cells, n (%) | 100/200 (50.0%) | 17/98 (17.4%) | 5/47 (10.7%) | 3/5 (60.0%) |
| Fresh frozen plasma,  n (%) | 89/200 (44.5%) | 11/98 (11.2%) | 7/47 (14.9%) | 2/5 (40.0%) |
| Platelets,  n (%) | 13/200 (6.5%) | 11/98 (11.2%) | 11/47 (23.4%) | 1/5 (20.0%) |
| Target haemoglobin value | - Hb target: 8 g/dL  - transfused to maintain  - measured every 30 min | - General Hb target ~ 8.5 g/dL (transfused to maintain)  - measured every 30 min | - Hb target: 7-9 g/dL  - transfused to maintain  - measured every 30 min | - Hb target: 8-9 g/dL  - transfused to maintain  - measured every 30 min |
| **Hemodynamic management** | **Center 1 (Berlin)** | **Center 2 (Edmonton)** | **Center 3 (Melbourne)** | **Center 4 (Dublin)** |
| Inotropes  (number of patients) | Inotropes day of surgery  n= 136/200  Dobutamine 12/136 [9%]  Milrinone 24/136 [18%]  Epinephrine 134/136 [99%]  Inotropes day 1  n=101/200  Dobutamine 13/101 [13%]  Milrinone 18/101 [18%]  Epinephrine 100/101 [99%]  Inotropes to achieve cardiac index >1.8-2.0 L/min/m^2^ | Inotropes day of surgery n=17/98  Dobutamine 8/17 [47%]  Milrinone 8/17 [47%]  Epinephrine 1/17 [6%]  Inotropes day 1  n=15/98  Dobutamine 9/15 [60%]  Milrinone 6/15 [40%]  Epinephrine 0/15 [0%]  Inotropes to achieve cardiac index >1.8-2.0 L/min/m^2^ | Inotropes day of surgery  n=12/47  Dobutamine 0/12 [0%]  Milrinone 12/12 [100%]  Epinephrine 2/12 [17%]  Inotropes day 1  n=10/47  Dobutamine 0/10 [0%]  Milrinone 8/10 [80%]  Epinephrine 2/10 [20%]  Milrinone to achieve cardiac index >2.0 L/min/m^2^ | Inotropes day of surgery  n=5/5    Dobutamine 0/0 [0%]  Milrinone 0/0 [0%]  Epinephrine 5/5 [100%]  Inotropes day 1  n=1/5  Dobutamine 0/0 [0%]  Milrinone 0/0 [0%]  Epinephrine 1/1 [100%]  Inotropes to achieve cardiac index >1.8-2.0 L/min/m^2^ |
| Vasopressors  (number of patients) | Vasopressors day of surgery n=145/200  Norepinephrine 142/145 [98%]  Dopamine 16/145 [11%]  Epinephrine 0/145 [0%]  Vasopressin 2/145 [14%]  Metaraminol 0/145 [0%]  Vasopressors day 1 n=25/200  Norepinephrine 23/25 [92%]  Dopamine 4/25 [16%]  Epinephrine 0/25 [0%]  Vasopressin 5/25 [20%] Metaraminol 0/25 [0%] | Vasopressors day of surgery n=74/98  Norepinephrine 73/74 [98.6%]  Dopamine 1/74 [1.4%]  Epinephrine 4/74 [5.4%]  Vasopressin 5/74 [6.8%]  Metaraminol 0/74 [0%]  Vasopressors day 1 n=24/98  Norepinephrine 22/24 [92%]  Dopamine 4/24 [8.3%]  Epinephrine 0/24 [0%]  Vasopressin 2/24 [8.3%] Metaraminol 0/24 [0%] | Vasopressors day of surgery n=40/47  Norepinephrine 32/40 [80%]  Dopamine 0/40 [1.4%]  Epinephrine 3/40 [8%]  Vasopressin 1/40 [2.5%]  Metaraminol 16/40 [40%]  Vasopressors day 1 n=23/47  Norepinephrine 22/23 [96%]  Dopamine 0/23 [0%]  Epinephrine 1/23 [4%]  Vasopressin 1/23 [4%]  Metaraminol 0/23 [0%] | Vasopressors day of surgery n=5/5  Norepinephrine 1/5 [20%]  Dopamine 0/5 [0%]  Epinephrine 4/5 [80%]  Vasopressin 0/5 [0%]  Metaraminol 0/5 [0%]  Vasopressors day 1 n=2/5  Norepinephrine 1/5 [20%]  Dopamine 0/5 [0%]  Epinephrine 1/5 [20%]  Vasopressin 0/5 [0%] Metaraminol 0/0 [0%] |

39
