# Supplementary material for: Prophylactic Perioperative Sodium Bicarbonate to Prevent Acute Kidney Injury Following Open Heart Surgery: A Multicenter Double-Blinded Randomized Controlled Trial
Source: PLoS Med. 2013 Apr 16;10(4):e1001426. doi: 10.1371/journal.pmed.1001426 (PMC3627643; doi:10.1371/journal.pmed.1001426)
Supplement: Table S2 — Major endpoints in post hoc subgroup analysis. (DOC) [file pmed.1001426.s002.doc]

| **Table S2.** Major endpoints in post-hoc subgroup analysis. | | | |
| --- | --- | --- | --- |
| **Patients with preoperative eGFR^*^****<60mL/min/1.73m^2^** | | | |
|  | **Sodium Bicarbonate**  (N=66) | **Sodium Chloride**  (N=44) | *p* |
| sCrea increase >25% or >44 µmol/L, n | 34 (51.5%) | 18 (40.9%) | 0.275 |
| RIFLE R or worse, n | 17 (25.8%) | 10 (22.7%) | 0.717 |
| RRT, n | 5 (7.6%) | 3 (6.8%) | 0.999 |
| Length of stay in hospital, days | 19.5 (8.8-30.5) | 21.0 (13.0-26.0) | 0.713 |
| Died in Hospital, n | 7 (10.6%) | 2 (4.6%) | 0.408 |
| **Patients from Australia, Canada and Ireland** (excluding German center) | | | |
|  | **Sodium Bicarbonate**  (N=74) | **Sodium Chloride**  (N=76) | *p* |
| sCrea increase >25% or >44 µmol/L, n | 42 (56.8%) | 44 (57.9%) | 0.888 |
| RIFLE R or worse, n | 26 (35.1%) | 22 (29.0%) | 0.417 |
| RRT, n | 0 (0%) | 1 (1.3%) | 0.999 |
| Length of stay in hospital, days | 7.0 (5.0-9.0) | 7.0 (6.0-9.0) | 0.914 |
| Died in Hospital, n | 1 (1.4%) | 0 (0%) | 0.495 |
| **Patients without non-study bicarbonate infusion** | | | |
|  | **Sodium Bicarbonate**  (N=141) | **Sodium Chloride**  (N=128) | *p* |
| sCrea increase >25% or >44 µmol/L, n | 63 (44.7%) | 41 (32.0%) | 0.033 |
| RIFLE R or worse, n | 32 (22.7%) | 17 (13.3%) | 0.046 |
| RRT, n | 4 (2.8%) | 4 (3.1%) | 0.999 |
| Length of stay in hospital, days | 17.0 (7.0-24.0) | 19.0 (7.0-25.0) | 0.498 |
| Died in Hospital, n | 7 (5.0%) | 1 (0.8%) | 0.069 |

For continuous variables, values denote median (25^th^ - 75^th^ percentiles) [Mann Whitney *U* test].

**^*^**eGFR, estimated glomerular filtration rate using the CKD-EPI formula [16]

sCrea, serum creatinine; RIFLE – Risk Injury Failure Loss and End stage renal failure-classification [15].

RRT, renal replacement therapy

39
