# Supplementary material for: Prophylactic Perioperative Sodium Bicarbonate to Prevent Acute Kidney Injury Following Open Heart Surgery: A Multicenter Double-Blinded Randomized Controlled Trial
Source: PLoS Med. 2013 Apr 16;10(4):e1001426. doi: 10.1371/journal.pmed.1001426 (PMC3627643; doi:10.1371/journal.pmed.1001426)
Supplement: Table S3 — Outcomes separated by study center. (DOC) [file pmed.1001426.s003.doc]

| **Table S3.** Outcomes separated by study center. | | | |
| --- | --- | --- | --- |
|  | **Sodium Bicarbonate** | **Sodium Chloride** | *p* |
| *Renal outcomes* |  |  |  |
| **Primary outcome^*^** |  |  |  |
| Center 1 (Berlin, Germany), n | 41/100 (41%) | 20/100 (20%) | 0.002 |
| Center 2 (Edmonton, Canada), n | 31/48 (64.6%) | 29/50 (58%) | 0.539 |
| Center 3 (Melbourne, Australia), n | 11/24 (45.8%) | 13/23 (56.5%) | 0.564 |
| Center 4 (Dublin, Ireland), n | 0/2 (0%) | 2/3 (66.7%) | 0.400 |
| **Secondary renal outcomes** |  |  |  |
| RIFLE AKI |  |  |  |
| Center 1 (Berlin, Germany), n | 16/100 (16%) | 9/100 (9%) | 0.199 |
| Center 2 (Edmonton, Canada), n | 15/48 (31.3%) | 14/50 (28%) | 0.826 |
| Center 3 (Melbourne, Australia), n | 10/24 (41.7%) | 7/23 (30.4%) | 0.547 |
| Center 4 (Dublin, Ireland), n | 0/2 (0%) | 1/3 (33.3%) | 0.999 |
| Increase in serum creatinine >50% |  |  |  |
| Center 1 (Berlin, Germany), n | 15/100 (15%) | 7/100 (7%) | 0.112 |
| Center 2 (Edmonton, Canada), n | 10/48 (20.8%) | 8/50 (16%) | 0.607 |
| Center 3 (Melbourne, Australia), n | 5/24 (20.8%) | 4/23 (17.4%) | 0.999 |
| Center 4 (Dublin, Ireland), n | 0/2 (0%) | 0/3 (0%) | 0.999 |
| Increase in serum creatinine >100% |  |  |  |
| Center 1 (Berlin, Germany), n | 4/100 (4%) | 4/100 (4%) | 0.999 |
| Center 2 (Edmonton, Canada), n | 3/48 (6.3%) | 1/50 (2%) | 0.357 |
| Center 3 (Melbourne, Australia), n | 0/24 (0%) | 0/23 (0%) | 0.999 |
| Center 4 (Dublin, Ireland), n | 0/2 (0%) | 0/3 (0%) | 0.999 |

^*^Serum creatinine increase as specified in the methods section.

39

| **Table S3 (cont.).** Outcomes separated by study center. | | | |
| --- | --- | --- | --- |
|  | **Sodium Bicarbonate** | **Sodium Chloride** | *p* |
| *Renal outcomes* |  |  |  |
| **Secondary renal outcomes** |  |  |  |
| ΔSerum creatinine, µmol/L |  |  |  |
| Center 1 (Berlin, Germany) | 17 (1-36) | 9 (0-20) | 0.012 |
| Center 2 (Edmonton, Canada) | 26 (16-39) | 24 (15-35) | 0.308 |
| Center 3 (Melbourne, Australia) | 21 (9-41) | 19 (13-30) | 0.958 |
| Center 4 (Dublin, Ireland) | 6 (2-7) | 34 (17-62) | 0.200 |
| Peak serum creatinine, µmol/L |  |  |  |
| Center 1 (Berlin, Germany) | 106 (88-150) | 97 (82-122) | 0.012 |
| Center 2 (Edmonton, Canada) | 110 (94-133) | 106 (96-125) | 0.447 |
| Center 3 (Melbourne, Australia) | 106 (76-143) | 88 (77-105) | 0.412 |
| Center 4 (Dublin, Ireland) | 94 (58-83) | 111 (94-209) | 0.400 |
| Postoperative acute renal replacement therapy |  |  |  |
| Center 1 (Berlin, Germany), n | 8/100 (8%) | 5/100 (5%) | 0.568 |
| Center 2 (Edmonton, Canada), n | 0/48 (0%) | 1/50 (2%) | 0.999 |
| Center 3 (Melbourne, Australia), n | 0/24 (0%) | 0/23 (0%) | 0.999 |
| Center 4 (Dublin, Ireland), n | 0/2 (0%) | 0/3 (0%) | 0.999 |

For continuous variables, values denote median (25^th^ - 75^th^ percentiles) [Mann Whitney *U* test].

| **Table S3 (cont.).** Outcomes separated by study center. | | | |
| --- | --- | --- | --- |
|  | **Sodium Bicarbonate** | **Sodium Chloride** | *p* |
| *Other outcomes* |  |  |  |
| **Urinary pH** |  |  |  |
| Preoperative |  |  |  |
| Center 1 (Berlin, Germany) | 5.0 (5.0-6.0) | 6.0 (5.0-6.5) | 0.013 |
| Center 2 (Edmonton, Canada) | 6.0 (5.5-7.0) | 6.0 (5.5-7.0) | 0.609 |
| Center 3 (Melbourne, Australia) | 5.0 (5.0-6.0) | 5.0 (5.0-6.0) | 0.882 |
| Center 4 (Dublin, Ireland) | 6.0 (5.0-6.0) | 6.0 (5.0-6.0) | 0.930 |
| 6 hours after CPB-Start |  |  |  |
| Center 1 (Berlin, Germany) | 6.5 (6.0-7.0) | 6.0 (5.0-6.5) | <0.001 |
| Center 2 (Edmonton, Canada) | 6.5 (5.5-7.5) | 5.5 (5.0-5.5) | <0.001 |
| Center 3 (Melbourne, Australia) | 7.0 (6.0-7.0) | 6.0 (5.0-7.0) | 0.026 |
| Center 4 (Dublin, Ireland) | 6.5 (6.0-6.5) | 6.5 (6.0-6.5) | 0.800 |
| 24 hours after CPB-Start |  |  |  |
| Center 1 (Berlin, Germany) | 7.5 (7.0-8.0) | 6.0 (5.0-7.0) | <0.001 |
| Center 2 (Edmonton, Canada) | 7.0 (5.5-8.0) | 5.5 (5.0-5.5) | <0.001 |
| Center 3 (Melbourne, Australia) | 7.0 (6.0-8.0) | 5.0 (5.0-6.0) | <0.001 |
| Center 4 (Dublin, Ireland) | 6.5 (5.5-7.0) | 6.0 (5.5-6.5) | 0.400 |

Values denote median (25^th^ - 75^th^ percentiles) [Mann Whitney *U* test].

| **Table S3 (cont.).** Outcomes separated by study center. | | | |
| --- | --- | --- | --- |
|  | **Sodium Bicarbonate** | **Sodium Chloride** | *p* |
| *Other outcomes* |  |  |  |
| **Plasma pH** |  |  |  |
| Preoperative |  |  |  |
| Center 1 (Berlin, Germany) | 7.41 (7.39-7.44) | 7.42 (7.39-7.44) | 0.298 |
| Center 2 (Edmonton, Canada) | 7.39 (7.36-7.43) | 7.39 (7.38-7.42) | 0.424 |
| Center 3 (Melbourne, Australia) | 7.39 (7.36-7.43) | 7.39 (7.36-7.42) | 0.653 |
| Center 4 (Dublin, Ireland) | 7.38 / 7.41^*^ | 7.40 (7.39-7.42) | 0.800 |
| 6 hours after CPB-Start |  |  |  |
| Center 1 (Berlin, Germany) | 7.43 (7.34-7.49) | 7.39 (7.33-7.44) | 0.017 |
| Center 2 (Edmonton, Canada) | 7.42 (7.37-7.46) | 7.37 (7.34-7.41) | 0.002 |
| Center 3 (Melbourne, Australia) | 7.43 (7.37-7.45) | 7.36 (7.33-7.41) | 0.013 |
| Center 4 (Dublin, Ireland) | 7.40 / 7.48^*^ | 7.38 (7.36-7.40) | 0.200 |
| 24 hours after CPB-Start |  |  |  |
| Center 1 (Berlin, Germany) | 7.46 (7.43-7.49) | 7.41 (7.39-7.43) | <0.001 |
| Center 2 (Edmonton, Canada) | 7.43 (7.40-7.45) | 7.37 (7.34-7.41) | <0.001 |
| Center 3 (Melbourne, Australia) | 7.44 (7.41-7.45) | 7.37 (7.33-7.40) | <0.001 |
| Center 4 (Dublin, Ireland) | 7.42 / 7.44^*^ | 7.34 (7.33-7.39) | 0.200 |

Values denote median (25^th^ - 75^th^ percentiles) [Mann Whitney *U* test]. ^*^Denotes individual patient data.

| **Table S3 (cont.).** Outcomes separated by study center. | | | |
| --- | --- | --- | --- |
|  | **Sodium Bicarbonate** | **Sodium Chloride** | *p* |
| *Other outcomes* |  |  |  |
| Length of ventilation, hours |  |  |  |
| Center 1 (Berlin, Germany) | 21 (14-49) | 22 (13-31) | 0.761 |
| Center 2 (Edmonton, Canada) | 15 (12-21) | 15 (12-20) | 0.887 |
| Center 3 (Melbourne, Australia) | 15 (12-19) | 13 (10-15) | 0.250 |
| Center 4 (Dublin, Ireland) | 16 (11-17) | 14 (14-18) | 0.800 |
| Length of stay in intensive care, hours |  |  |  |
| Center 1 (Berlin, Germany) | 30 (23-124) | 27 (22-73) | 0.118 |
| Center 2 (Edmonton, Canada) | 41 (23-90) | 43 (24-91) | 0.672 |
| Center 3 (Melbourne, Australia) | 26 (20-49) | 23 (20-44) | 0.602 |
| Center 4 (Dublin, Ireland) | 60 (17-74) | 22 (21-116) | 0.800 |
| Length of stay in hospital, days |  |  |  |
| Center 1 (Berlin, Germany) | 23 (19-33) | 23 (19-30) | 0.514 |
| Center 2 (Edmonton, Canada) | 6 (5-8) | 7 (5-8) | 0.521 |
| Center 3 (Melbourne, Australia) | 8 (7-10) | 7 (6-10) | 0.433 |
| Center 4 (Dublin, Ireland) | 12 (7-13) | 16 (8-22) | 0.667 |
| Died in hospital |  |  |  |
| Center 1 (Berlin, Germany), n | 10/100 (10%) | 3/100 (3%) | 0.082 |
| Center 2 (Edmonton, Canada), n | 0/48 (0%) | 0/50 (0%) | 0.999 |
| Center 3 (Melbourne, Australia), n | 1/24 (4.2%) | 0/23 (0%) | 0.999 |
| Center 4 (Dublin, Ireland), n | 0/2 (0%) | 0/3 (0%) | 0.999 |

For continuous variables, values denote median (25^th^ - 75^th^ percentiles) [Mann Whitney *U* test].
