# Supplementary material for: Prophylactic Perioperative Sodium Bicarbonate to Prevent Acute Kidney Injury Following Open Heart Surgery: A Multicenter Double-Blinded Randomized Controlled Trial
Source: PLoS Med. 2013 Apr 16;10(4):e1001426. doi: 10.1371/journal.pmed.1001426 (PMC3627643; doi:10.1371/journal.pmed.1001426)
Supplement: Text S1 — Trial protocol. (DOC) [file pmed.1001426.s004.doc]

# Sodium Bicarbonate in Cardiac Surgery Study (Bic-MC)

This study has been terminated.

Sponsor:

Austin Health

Information provided by (Responsible Party):

Rinaldo Bellomo, Austin Health

ClinicalTrials.gov Identifier:

NCT00672334

First received: May 1, 2008

Last updated: July 31, 2012

Last verified: July 2012

| **Tracking Information** | |
| --- | --- |
| **First Received Date  ^ICMJE^** | May 1, 2008 |
| **Last Updated Date** | July 31, 2012 |
| **Start Date  ^ICMJE^** | May 2008 |
| **Primary Completion Date** | June 2011   (final data collection date for primary outcome measure) |
| **Current Primary Outcome Measures  ^ICMJE^   (submitted: June 21, 2011)** | Proportion of patients developing an increase in serum creatinine > 25% or >44µmicromol/L from baseline to peak level after adjustment for relevant baseline characteristics [ Time Frame: within first two-five postoperative days. ] [ Designated as safety issue: No ] |
| **Original Primary Outcome Measures  ^ICMJE^   (submitted: May 1, 2008)** | Proportion of patients developing an increase in serum creatinine > 25% or >44µmicromol/L from baseline to peak level [ Time Frame: within first two-five postoperative days. ] [ Designated as safety issue: No ] |
| **Change History** | [Complete list of historical versions of study NCT00672334 on ClinicalTrials.gov Archive Site](http://www.clinicaltrials.gov/ct2/archive/NCT00672334) |
| **Current Secondary Outcome Measures  ^ICMJE^   (submitted: June 21, 2011)** | - Proportion of patients developing an increase in serum creatinine greater than 50% from baseline to peak level after adjustment for relevant baseline characteristics [ Time Frame: within first two-five postoperative days ] [ Designated as safety issue: No ] - Proportion of patients developing an increase in serum creatinine greater than 100% from baseline to peak level after adjustment for relevant baseline characteristics [ Time Frame: within first two-five postoperative days ] [ Designated as safety issue: No ] - Change in serum creatinine from baseline to peak level after adjustment for relevant baseline characteristics [ Time Frame: within first two-five postoperative days ] [ Designated as safety issue: No ] - Proportion of patients developing any of the RIFLE criteria: R, I or F after adjustment for relevant baseline characteristics [ Time Frame: within first five postoperative days ] [ Designated as safety issue: No ] - Proportion of patients developing any of the AKI stages: 1, 2 or 3 (using network definition)after adjustment for relevant baseline characteristics [ Time Frame: within 48 hours postoperatively ] [ Designated as safety issue: No ] - Change in serum urea from baseline to peak [ Time Frame: within first two-five postoperative days ] [ Designated as safety issue: No ] - Change in NGAL from baseline to peak [ Time Frame: within first 24 postoperatively ] [ Designated as safety issue: No ] - Change in electrolyte status from baseline to peak [ Time Frame: within first 24-48hrs postoperatively ] [ Designated as safety issue: Yes ] - Requirement of renal replacement therapy [ Time Frame: within first postoperative days ] [ Designated as safety issue: No ] - Length of ventilation [ Time Frame: from commencement to end of intubation ] [ Designated as safety issue: No ] - Length of stay in Intensive care [ Time Frame: from admission to discharge from Intensive care ] [ Designated as safety issue: No ] - Length of stay in hospital [ Time Frame: from admission to discharge from hospital ] [ Designated as safety issue: No ] - Hospital-Mortality [ Time Frame: during hospital stay ] [ Designated as safety issue: No ] - 90-day mortality [ Time Frame: during 90 days postoperatively ] [ Designated as safety issue: No ] - COMT polymorphism [ Time Frame: sampling at induction of anesthesia ] [ Designated as safety issue: No ] |
| **Original Secondary Outcome Measures  ^ICMJE^   (submitted: May 1, 2008)** | - Proportion of patients developing an increase in serum creatinine greater than 50% from baseline to peak level [ Time Frame: within first two-five postoperative days ] [ Designated as safety issue: No ] - Proportion of patients developing an increase in serum creatinine greater than 100% from baseline to peak level. [ Time Frame: within first two-five postoperative days ] [ Designated as safety issue: No ] - Change in serum creatinine from baseline to peak level [ Time Frame: within first two-five postoperative days ] [ Designated as safety issue: No ] - Proportion of patients developing any of the RIFLE criteria: R, I or F [ Time Frame: within first five postoperative days ] [ Designated as safety issue: No ] - Proportion of patients developing any of the AKI stages: 1, 2 or 3 (using network definition) [ Time Frame: within 48 hours postoperatively ] [ Designated as safety issue: No ] - Change in serum urea from baseline to peak [ Time Frame: within first two-five postoperative days ] [ Designated as safety issue: No ] - Change in NGAL from baseline to peak [ Time Frame: within first 24 postoperatively ] [ Designated as safety issue: No ] - Change in electrolyte status from baseline to peak [ Time Frame: within first 24-48hrs postoperatively ] [ Designated as safety issue: Yes ] - Requirement of renal replacement therapy [ Time Frame: within first postoperative days ] [ Designated as safety issue: No ] - Length of ventilation [ Time Frame: from commencement to end of intubation ] [ Designated as safety issue: No ] - Length of stay in Intensive care [ Time Frame: from admission to discharge from Intensive care ] [ Designated as safety issue: No ] - Length of stay in hospital [ Time Frame: from admission to discharge from hospital ] [ Designated as safety issue: No ] - Hospital-Mortality [ Time Frame: during hospital stay ] [ Designated as safety issue: No ] - 90-day mortality [ Time Frame: during 90 days postoperatively ] [ Designated as safety issue: No ] |
| **Current Other Outcome Measures  ^ICMJE^** |  |
| **Original Other Outcome Measures  ^ICMJE^** |  |
|  |  |
| **Descriptive Information** | |
| **Brief Title  ^ICMJE^** | Sodium Bicarbonate in Cardiac Surgery Study |
| **Official Title  ^ICMJE^** | A Multicenter, Randomized, Double Blind, Placebo Controlled Study of the Effect of Sodium Bicarbonate on Postoperative Renal Function in Patients Undergoing Elective Cardiopulmonary Bypass |
| **Brief Summary** | With over one million operations a year, cardiac surgery with cardiopulmonary bypass is one of the most common major surgical procedures worldwide (1). Acute kidney injury is a common and serious postoperative complication of cardiopulmonary bypass and may affect 25% to 50% of patients (2-4). Acute kidney injury carries significant costs (4) and is independently associated with increased morbidity and mortality (2,3). Even minimal increments in plasma creatinine are associated with an increase in mortality (5,6).  Multiple causes of cardiopulmonary bypass-associated acute kidney injury have been proposed, including ischemia-reperfusion, generation of reactive oxygen species, hemolysis and activation of inflammatory pathways (7-10). COMT LL genotype appears to increase the risk of vasodilatory shock and AKI after cardiac surgery. To date, no simple, safe and effective intervention to prevent cardiopulmonary bypass-associated acute kidney injury in a broad patient population has been found (11-14).  Urinary acidity may enhance the generation and toxicity of reactive oxygen species induced by cardiopulmonary bypass (10,15). Activation of complement during cardiac surgery (16) may also participate in kidney injury. Urinary alkalinization may protect from kidney injury induced by oxidant substances, iron-mediated free radical pathways, complement activation and tubular hemoglobin cast formation (9,17,18). Of note, increasing urinary pH - in combination with N-acetylcysteine (19,20) or without (21) - has recently been reported to attenuate acute kidney injury in patients undergoing contrast-media infusion.  In a pilot double-blind, randomized controlled trial the investigators found sodium bicarbonate to be efficacious, safe, inexpensive and easy to administer. These findings now need to be confirmed or refuted by further clinical investigations in other geographic and institutional settings.  Accordingly, the investigators hypothesized that urinary alkalinization might protect kidney function in patients at increased risk of acute kidney injury undergoing cardiopulmonary bypass needs to be confirmed in an international multicenter, double-blind, randomized controlled trial of intravenous sodium bicarbonate. |
| **Detailed Description** | Renal impairment following cardiopulmonary bypass is common. While most of these patients do not require either short or long term renal replacement, the mortality of patients with acute renal failure is substantially greater than those who do not develop renal dysfunction.  In a pilot double-blind, randomized controlled trial we found sodium bicarbonate to be efficacious, safe, inexpensive and easy to administer. These findings now need to be confirmed or refuted by further clinical investigations in other geographic and institutional settings.  There is evidence that sodium bicarbonate affects the cardiovascular, respiratory and immune systems and may be of benefit to patients undergoing cardiac surgery.  Study Design - overview and rationale Patients will be randomised to receive sodium bicarbonate from the induction of anaesthesia until 24 hours postoperatively, or a placebo (sodium chloride).  Serum creatinine is the most commonly used clinical indicator of renal function along with urine output. Both will be measured for several days postoperatively - the time period during which renal impairment is most likely to develop.  Randomisation The randomisation will be based on random numbers generated by computer. Once consent is obtained, the allocation of either treatment with sodium bicarbonate or placebo will be organised by an independent person (clinical trials pharmacist) who will dispense the coded and blinded infusion bags (shrink-wrapped in extra black plastic bags). This will be delivered to the anaesthetic staff looking after the patient in theatre, and the ICU nurse caring for the patient postoperatively.  20 ml samples of heparinised blood and urine will be taken from the arterial line or urine catheter. Samples will be taken immediately after the preoperative insertion of the arterial/urine catheter, at 6, 24, 48, 72, 96 and 120 hours after commencement of cardiopulmonary bypass. Immediately following collection, the preoperative, 6 and 24 hour blood and urine will be centrifuged at low speed to separate the plasma from the cellular components. Urine and plasma and full-blood (for COMT polymorphism) will be stored in aliquots (where necessary) at -70 degrees prior to batch analysis.  The following variables will be obtained:  Code for patient, gender and age. Date and time of admission to ICU Operative procedure and date and time on and off cardiopulmonary bypass Preoperative assessment of left ventricular function, Comorbidities, Pre-, intra- and post-operative medication, Markers of renal function and COMT polymorphism as described above, Doses of frusemide administered (or rate of frusemide infusion) Use of inotropes or vasopressors Cardiac output whenever measured for clinical purposes in the first 24 hours postoperatively Requirement of renal replacement therapy Urine output in each 6 hour period during the presence of urine catheter Acid base status and electrolytes at baseline, 6 and 24 hours after commencement of cardiopulmonary bypass, Time of intubation and extubation, Date and time of arrival on and discharge from ICU and hospital, death Resources required The principle of the study has been discussed with the involved cardiac anaesthetists, cardiac surgeons, intensivists and intensive care nurses, who have offered their co-operation. ICU research nurse to allocate patients and collect clinical data. Pharmacy will be required to prepare drug and placebo infusion bags. Clinical pathology will be required to perform 24 hour creatinine clearance estimation (in addition to those tests clinically indicated) Protocol violations All protocol violations will be recorded. It will then be decided whether the nature of such violation had been such that the patient should be excluded from primary data analysis. Such evaluation will be blinded to treatment.  Withdrawal The treating clinician will have the right to withdraw the patient from the study if he or she believes that continued participation is jeopardising the patient's well being.  Ethical Issues sodium bicarbonate used in this study is considered to be very safe as has been demonstrated by its widespread clinical use in the management of critically ill patients with metabolic acidosis. We consider the potential benefit of this treatment theoretically significant. Given the balance of benefits and risks, we consider it ethical to proceed and seek informed consent.  Indemnity This is an investigator-initiated study and, accordingly, no commercial sponsor's indemnity has been provided.  Informed consent will be obtained from the patient prior to the operation by one of the investigators or the ICU research nurse. The clinical care of a patient who does not consent for any reason will not be affected. |
| **Study Type  ^ICMJE^** | Interventional |
| **Study Phase** | Phase 2 Phase 3 |
| **Study Design  ^ICMJE^** | Allocation: Randomized Endpoint Classification: Safety/Efficacy Study Intervention Model: Parallel Assignment Masking: Double Blind (Subject, Caregiver, Investigator, Outcomes Assessor) Primary Purpose: Prevention |
| **Condition  ^ICMJE^** | - Cardiac Surgery - Cardiopulmonary Bypass |
| **Intervention  ^ICMJE^** | - Drug: Sodium Bicarbonate   In all patients body weight adjusted dose of study medication will be achieved by infusion of sodium bicarbonate at a dose of 0.5 mmol/kg body weight (=bolus) diluted in 250 mL over 1 hour immediately after the induction of anesthesia, prior to the first surgical incision followed by continuous intravenous infusion of 0.2 mmol/kg/hr (=maintenance) diluted in 1000 mL 23 hours (total dose of 5 mmol/kg over 24 hours).  Other Name: Hypertonic bicarbonate   - Drug: Sodium Chloride   In all patients body weight adjusted dose of study medication will be achieved by infusion of sodium chloride at a dose of 0.5 mmol/kg body weight (=bolus) diluted in 250 mL over 1 hour immediately after the induction of anesthesia, prior to the first surgical incision followed by continuous intravenous infusion of 0.2 mmol/kg/hr (=maintenance) diluted in 1000 mL 23 hours (total dose of 5 mmol/kg over 24 hours).  Other Name: hyeprtonic sodium chloride |
| **Study Arm (s)** | - Placebo Comparator: 2   sodium chloride at 0.5 mmol/kg loading pre-induction and then at 0.2 mmol/kg/hr over 24 hours after induction until the next day  Intervention: Drug: Sodium Chloride   - Experimental: 1   The active intervention is loading (05. mmol/kg) pre-surgery and continuous infusion of bicarbonate at 0.2 mmol/kg/hr for 24 hours after induction  Intervention: Drug: Sodium Bicarbonate |
| **Publications *** |  |
| *   Includes publications given by the data provider as well as publications identified by ClinicalTrials.gov Identifier (NCT Number) in Medline. | |
|  |  |
| **Recruitment Information** | |
| **Recruitment Status  ^ICMJE^** | Terminated |
| **Enrollment  ^ICMJE^** | 350 |
| **Completion Date** | January 2012 |
| **Primary Completion Date** | June 2011   (final data collection date for primary outcome measure) |
| **Eligibility Criteria  ^ICMJE^** | Inclusion Criteria:   - Cardiac surgical patients in whom the use of cardiopulmonary bypass was planned and: - Written informed consent of patient - Age >18 years - And having at least one ore more of the following risk factors for postoperative AKI:   - Age =/>70 years   - Preoperative plasma creatinine >120 µmol/L New York Heart Association class III / IV or LVEF <35%   - Insulin dependent diabetes mellitus   - Valve surgery (with or without coronary artery bypass graft)   - Redo cardiac surgery   Exclusion Criteria:   - Cardiac surgical patients will not be considered eligible if: - An emergency operation is indicated (within 24 hours after hospital admission or on intra-aortic balloon pump) or - Pregnancy is confirmed or breastfeeding is present or - A renal allograft is present or - Preoperative acute renal failure within 6 weeks (acute rise in serum creatinine >50% from baseline) is present or - Pre-operative end stage renal disease (serum creatinine >300 µmol/L) is present or - Chronic moderate to high dose corticosteroid therapy (>10 mg/d prednisone or equivalent) is present |
| **Gender** | Both |
| **Ages** | 18 Years and older |
| **Accepts Healthy Volunteers** | No |
| **Contacts  ^ICMJE^** | *Contact information is only displayed when the study is recruiting subjects* |
| **Location Countries  ^ICMJE^** | Australia,   Canada,   Germany,   Ireland |
|  |  |
| **Administrative Information** | |
| **NCT Number  ^ICMJE^** | NCT00672334 |
| **Other Study ID Numbers  ^ICMJE^** | EudraCT 2007-002223-32 |
| **Has Data Monitoring Committee** | Yes |
| **Responsible Party** | Rinaldo Bellomo, Austin Health |
| **Study Sponsor  ^ICMJE^** | Austin Health |
| **Collaborators  ^ICMJE^** |  |
| **Investigators  ^ICMJE^** | | Study Chair: | Rinaldo Bellomo, MD, FRACP | Austin Health, Melbourne, Australia |  | | --- | --- | --- | --- | | Principal Investigator: | Michael Haase, MD | Charité University Medicine Berlin, Germany |  | | Principal Investigator: | Sean M Bagshaw, MD | University of Alberta, Edmonton, Canada |  | | Principal Investigator: | Patrick Murray, MD | University Clinic Dublin, Dublin, Ireland |  | |
| **Information Provided By** | Austin Health |
| **Verification Date** | July 2012 |
